# ACE2 knockout hinders SARS-CoV-2 propagation in iPS cell-derived airway and alveolar epithelial cells

**DOI:** 10.3389/fcell.2023.1290876

**Published:** 2023-12-08

**Authors:** Ryo Niwa, Kouji Sakai, Mandy Siu Yu Lung, Tomoko Matsumoto, Ryuta Mikawa, Shotaro Maehana, Masato Suzuki, Yuki Yamamoto, Thomas L. Maurissen, Ai Hirabayashi, Takeshi Noda, Makoto Kubo, Shimpei Gotoh, Knut Woltjen

**Affiliations:** ^1^ Department of Life Science Frontiers, Center for iPS Cell Research and Application (CiRA), Kyoto University, Kyoto, Japan; ^2^ Graduate School of Medicine, Kyoto University, Kyoto, Japan; ^3^ Department of Veterinary Science, National Institute of Infectious Diseases, Tokyo, Japan; ^4^ Management Department of Biosafety, Laboratory Animal, and Pathogen Bank, National Institute of Infectious Diseases, Tokyo, Japan; ^5^ Department of Clinical Application, Center for iPS Cell Research and Application (CiRA), Kyoto University, Kyoto, Japan; ^6^ Department of Microbiology, Kitasato University School of Allied Health Sciences, Kanagawa, Japan; ^7^ Regenerative Medicine and Cell Design Research Facility, Kitasato University School of Allied Health Sciences, Kanagawa, Japan; ^8^ Antimicrobial Resistance Research Center, National Institute of Infectious Diseases, Tokyo, Japan; ^9^ Laboratory of Ultrastructural Virology, Institute for Life and Medical Sciences, Kyoto University, Kyoto, Japan; ^10^ Laboratory of Ultrastructural Virology, Graduate School of Biostudies, Kyoto University, Kyoto, Japan

**Keywords:** iPS cells, CRISPR-Cas9, gene editing, gene knockout, ACE2, SARS-CoV-2

## Abstract

Severe Acute Respiratory Syndrome Coronavirus 2 (SARS-CoV-2), the causative agent of COVID-19, continues to spread around the world with serious cases and deaths. It has also been suggested that different genetic variants in the human genome affect both the susceptibility to infection and severity of disease in COVID-19 patients. Angiotensin-converting enzyme 2 (ACE2) has been identified as a cell surface receptor for SARS-CoV and SARS-CoV-2 entry into cells. The construction of an experimental model system using human iPS cells would enable further studies of the association between viral characteristics and genetic variants. Airway and alveolar epithelial cells are cell types of the lung that express high levels of ACE2 and are suitable for *in vitro* infection experiments. Here, we show that human iPS cell-derived airway and alveolar epithelial cells are highly susceptible to viral infection of SARS-CoV-2. Using gene knockout with CRISPR-Cas9 in human iPS cells we demonstrate that ACE2 plays an essential role in the airway and alveolar epithelial cell entry of SARS-CoV-2 *in vitro*. Replication of SARS-CoV-2 was strongly suppressed in ACE2 knockout (KO) lung cells. Our model system based on human iPS cell-derived lung cells may be applied to understand the molecular biology regulating viral respiratory infection leading to potential therapeutic developments for COVID-19 and the prevention of future pandemics.

## Introduction

Combining genome editing technology with human induced pluripotent stem (iPS) cells allows for differentiation of gene-edited iPS cells into functional somatic cell types, enabling studies of how genetic variations link genotype and phenotype (Okano and Yamanaka, 2014; Hockemeyer and Jaenisch, 2016; Ben Jehuda et al., 2018). CRISPR-Cas9 is the most widely used technology for gene editing (Wang and Doudna, 2023). The Cas9 nuclease is directed to its target site by a guide RNA (gRNA) consisting of 20 nucleotides that determines the position of double-strand break (DSB) formation in the target genome. The host cell then employs one of three major DNA repair pathways: non-homologous end joining (NHEJ), microhomology-mediated end joining (MMEJ), and homology directed repair (HDR) to mend the DSB (Yeh et al., 2019; Nambiar et al., 2022). NHEJ is typically attributed to the formation of random insertions and deletions (indels) and is commonly employed for gene knockout (KO). MMEJ produces deletions between short repeat sequences, or microhomologies, such that the size of the deletion can be reliably predicted (Ata et al., 2018; Shen et al., 2018; Martínez-Gálvez et al., 2021). Therefore, MMEJ has proven to be useful for reproducibly creating mutations that resulting in frameshifts and gene KO (Martínez-Gálvez et al., 2021), or naturally occurring pathogenic deletion variants (Ata et al., 2018; Grajcarek et al., 2019).

COVID-19, declared a pandemic in 2020, has drastically increased the number of deaths associated with infection throughout 2020 and 2021 (Forchette et al., 2021; Wang et al., 2022a), and its impact on global mortality rates and reduced life expectancy continues to this day. Molecular pathways for SARS-CoV-2 viral infection and the susceptible somatic cell types are gradually being uncovered, with two major pathways suggesting cell surface entry and endosomal entry (Scialo et al., 2020; Shang et al., 2020). Both pathways use Angiotensin Converting Enzyme 2 (ACE2) as a cell surface receptor. ACE2 serves to maintain the balance of angiotensin II (Ang II) levels in the bloodstream in the human body (Turner, 2015). Cell surface entry by SARS viruses is achieved by Spike protein (S-protein) priming by TMPRSS2, followed by binding to ACE2. On the other hand, SARS viruses can enter the cell via a TMPRSS2-independent endosomal entry pathway (Kimura et al., 2022; Meng et al., 2022; Peacock et al., 2022; Willett et al., 2022). It is essential to build *in vitro* models that recapitulate the physiological effects of viral infection to unravel the mechanisms of infection at the molecular level.

As reported previously, infection models using iPS cell-derived airway and alveolar epithelial cells are useful systems for this purpose (Huang et al., 2020; Abo et al., 2022; Wang et al., 2022b; Kimura et al., 2022; Saito et al., 2022). In lung tissue, the airways and alveoli are the main target tissues of SARS-CoV-2 and creating them *in vitro* using gene-edited iPS cells would allow for a detailed evaluation of viral pathology. SARS-CoV-2 has been analyzed using iPS cell-derived alveolar and airway epithelial cells based on air-liquid interface (ALI) culture (Kimura et al., 2022; Saito et al., 2022). ACE2 is expressed in these cells (Hikmet et al., 2020; Yao et al., 2020; Zhao et al., 2020) and therefore a clear demonstration of ACE2-mediated viral entry in iPS cell-derived models would aid in various aspects of viral biology, including the evaluation of viral variants.

In this study, we created ACE2 KO iPS cells with high efficiency and reproducibility by designing gRNAs likely to result in predictable MMEJ deletions (Shang et al., 2020). ACE2-deficiency showed little effect on the differentiation or integrity of lung epithelial cells. We subsequently established an iPS cell-derived model of lung infection and demonstrated that ACE2 is essential for efficient SARS-CoV-2 viral entry and replication. Our study demonstrates that an *in vitro* system can mimic SARS-CoV-2 entry into lung cell types as observed *in vivo*. Differentiation of gene-edited iPS cell lines presents a valuable tool to predict the role of genes and gene variants related to viral infection, which would greatly contribute to infectious disease prevention and treatment.

## Materials and methods

### Culture of human iPS cells

The B2-3 lung reporter iPS cell line (Gotoh et al., 2014) was maintained at 37 °C and 5% CO_2_ in StemFit AK02N medium (Ajinomoto, Cat. No. RCAK02N) on tissue culture plates coated with 0.5 mg/mL silk iMatrix-511, Recombinant Human Laminin-511 E8 Fragment (Nippi, Cat. No. 892021) with a daily medium exchange (Gotoh et al., 2014). Cell passage was performed every 7 days during maintenance. Cells were first dissociated with Accumax (Innovative Cell Technologies, Cat. No. AM105-500) and 10 min incubation at 37 °C, then washed in StemFit AK02N medium supplemented with 10 µM ROCK inhibitor Y-27632 (Wako, Cat. No. 253-00513) and seeded onto iMatrix511-coated plates at a density of 1  ×  10^3^ cells/cm^2^ in StemFit AK02N medium with ROCK inhibitor for 48 h after seeding, and then cultured without ROCK inhibitor. All the cell lines were routinely tested as negative for *mycoplasma* contamination.

### Guide RNA design

Guide RNA (gRNA) was designed with MMEJ kNockout Target Heuristic Utility (MENTHU) web tool version (Ata et al., 2018). After inputting the genbank ID of ACE2 gene (NG_012575.3), we filtered the list of gRNAs based on a MENTHU score >2.0. The sequence of guide RNA is listed in Supplementary Table S1.

### Gene editing of iPS cells

Gene editing experiments were performed as previously described (Maurissen and Woltjen, 2020). Briefly, an equimolar amount of crRNA and tracrRNA sequences (IDT, Alt-R CRISPR-Cas9 crRNA and tracrRNA) were hybridized for 5 min at 95°C to form functional crRNA:tracrRNA duplexes (gRNA). For each electroporation, 61 pmol gRNA was mixed with 61 pmol Cas9 nuclease (IDT, Alt-R S.p. Cas9 Nuclease V3) (1:1 gRNA:Cas9 ratio) to form RNP complexes, and incubated for 30 min at room temperature (RT). gRNAs are listed in Supplementary Table S1. Cells were harvested with Accumax, washed, counted, and resuspended at a density of 5  ×  10^5^ cells/10 µL in Opti-MEM I reduced-serum medium (Life Technologies, Cat. No. 31985-062). 5  ×  10^5^ cells were added to the RNP mixture and a total volume of 50 µL was electroporated in a Nepa Electroporation Cuvette 1 mm gap (Nepa Gene, Cat. No. EC-001) using the NEPA21 Electroporator (Nepa Gene) instrument (Poring pulse: 125 V voltage, 2.5 ms pulse length, 50 ms pulse gap, 2 pulses, 10% pulse decay, +orientation; Transfer pulse: 20 V voltage, 50 ms pulse length, 50 ms pulse gap, 5 pulses, 40% pulse decay, ±orientation). Electroporated cells were then transferred to an iMatrix511-coated plate in StemFit AK02N medium supplemented with ROCK inhibitor and incubated at 37 °C, or at 32°C for 48 h for cold shock treatment and then incubated at 37°C. Medium exchange was performed after 48 h with StemFit AK02N without ROCK inhibitor, and cells were maintained normally until genome extraction and passaging.

### Genotyping of gene edited iPS cells

For genomic DNA extraction, 0.5–1  ×  10^6^ cells were washed with 1X DPBS, DNA was purified using the DNeasy Blood & Tissue Kit (Qiagen, Cat. No. 69506) as recommended by manufacturer’s instructions, and purified DNA was resuspended in 100 µL of water. Target sequences were amplified using KAPA HiFi HS ReadyMix (Kapa Biosystems, Cat. No. KK2602) polymerase chain reaction (PCR), enzymatic PCR product cleanup was performed with ExoSAP-IT Express reagent (Thermo Fischer Scientific, Cat. No. 75001), and Sanger sequencing was performed using the BigDye Terminator v3.1 CS Kit (Thermo Fischer Scientific, Cat. No. 4337456) according to the manufacturer’s instructions. Genotyping primers are listed in Supplementary Table S2. Reactions were then purified by ethanol precipitation and sequenced on a 3,500xl Genetic Analyzer (Applied Biosystems). Sequence alignments were analyzed with Snapgene (GSL Biotech LLC).

Detection of off-target (OT) sites was conducted with the API service of GGGenome (https://gggenome.dbcls.jp/en/). Genomic sites with mismatches of 3 bp or less across the 20 nt spacer region and no more than 1 bp mismatch in the seed sequence (the first 12 nt of the spacer) were considered potential OT sites based on GRCh38. OT sites were ranked by overlap with genomic features (genes, enhancers, and mammalian conservation). After PCR amplification, we confirmed the integrity of OT sites by alignment with sequence results from unedited B2-3 genomic DNA samples. Karyotyping was performed by G-band analysis (Nihon Gene Research Laboratories, Japan). Primers used for OT detection are shown in Supplementary Table S2.

### Immunostaining for pluripotency

Cells were fixed in 4% Paraformaldehyde in PBS without Mg^2+^ and Ca^2+^ (PBS^−^) for 10 min at room temp. Before immunostaining, the cells were treated with 0.1 M Glycine in PBS^−^ for 30 min and rinsed with PBS-. To perform NANOG and OCT3/4 staining, the cells were permeabilized with 0.1% Triton X-100 in PBS^−^ for 10 min at room temperature. The samples were blocked with 3% BSA in PBS^−^ for 60 min at room temperature and incubated overnight at 4°C with primary antibodies diluted in blocking solution. Antibody dilutions were as follows: anti-Nanog (CST Cat. 4903S) 1:500, anti-OCT3/4 (BD Pharmingen 611203) 1:250, anti-TRA-1-60 (BD Pharmingen 560071) 1:250; and anti-TRA-1-81 (BD Pharmingen 560072) 1:250. Samples were rinsed with PBS^−^ and incubated for 60 min at room temperature with secondary antibodies diluted 1:300 in blocking solution (Alexa-488 conjugated anti-rabbit IgG, anti-mouse IgG or anti-mouse IgM; ThermoFisher Scientific). DAPI 1 μg/mL was included together with the secondary antibodies. After rinsing with PBS^−^ images were acquired using a fluorescence microscope Keyence BZ-X700 with 10x lenses. Images were prepared using Adobe Photoshop and Illustrator software.

### Generation of airway and alveolar epithelial cells

Both airway and alveolar epithelial cells were differentiated from B2-3 SFTPC-GFP reporter (wild type) iPS cells or gene-edited ACE2 KO iPS cells via NKX2-1^+^ lung progenitor cells, respectively (Gotoh et al., 2014; Konishi et al., 2016; Yamamoto et al., 2017). In brief, iPS cells were stepwise differentiated into definitive endodermal cells, anterior foregut endodermal cells, and NKX2-1^+^ lung progenitor cells, followed by cell sorting using anti-carboxypeptidase M (CPM) antibody, as previously described (Yamamoto et al., 2017). Upon airway cell induction, isolated CPM^+^ cells were seeded onto iMatrix-511 silk (Takara Bio) (20 μg/cm^3^)-coated 24-well cell culture inserts (Falcon) at a density of 1.8 × 10^6^ cells/cm^2^ with PneumaCult-ALI Maintenance medium (STEMCELL Technologies) supplemented with 10 µM Y-27632 (LC Laboratories), 4 µg/mL heparin (Nacalai Tesque) and 1 µM hydrocortisone (Sigma-Aldrich). After the cells became confluent, the medium was further supplemented with 10 µM DAPT (Wako) and replenished every 2–7 days for a period of 1 month. Upon alveolar epithelial cell induction, isolated CPM^+^ lung progenitor cells were seeded onto Geltrex (Gibco)-coated 24-well cell culture inserts at a density of 8.3 × 10^5^ cells/cm^2^ and the cells were cultured for 1-week in ALI in fibroblast-free alveolarization medium exchanged every other day, as described previously (Yamamoto et al., 2017).

### RNA sequencing

To extract total RNA, we used the RNeasy Mini Kit following the manufacturer’s instructions. For preparing sequencing libraries, we used the TruSeq Stranded Total RNA kit from Illumina. The library was sequenced using the NovaSeq SP Reagent Kit v1.5 (200 cycles), with 101-8-8-101 cycles. We quantified gene expression using the analysis pipeline (2.3.4) described in the ENCODE project (https://www.encodeproject.org/pipelines/ENCPL002LPE/), using GRCh38 ENSEMBL release 98 as a reference sequence and GRCh38 GENCODE release 32 for gene definitions. Bar charts were produced using ggplot2 v3.4.2, ggprism v1.0.4 (https://cran.r-project.org/package=ggprism), and R version 4.1.2, based on a TPM-corrected expression matrix. We extracted genes highly characterized in lung tissue from the top 100 entries in High-expression in GTEx (Lonsdale et al., 2013; Ardlie et al., 2015) using Hamornizone 3.0 (Rouillard et al., 2016) and LungMAP (Sun et al., 2022).

### Western blot analysis

Harvested cells were rinsed with PBS before resuspending in RIPA buffer (Sigma, #R0278) containing 1X protease inhibitor cocktail made by dissolving cOmplete tablets (Roche, #04-693-159-001) in Milli-Q. Protein samples were sonicated and measured by BCA assay (Thermo Scientific, #23250), and 5–10 µg of total protein lysate was applied to each lane. Prior to loading, protein samples were mixed with a final concentration of 1X NuPAGE LDS Sample Buffer (Invitrogen, #NP0007) and 50 mM DTT, then heated at 70°C for 10 min to denature secondary protein structures and placed on ice until ready to load. Polyacrylamide gels were run in denaturing conditions using NuPAGE 10% Bis-Tris protein gels (Invitrogen, #NP0316BOX) and 1X MOPS (50 mM MOPS, 0.1% SDS, 50 mM Tris-Base, 1 mM EDTA) as running buffer. NuPAGE Antioxidant (Invitrogen, #NP0005) was added to the running buffer in 1/400 concentration to prevent sample reoxidation and to maintain proteins in a reduced state during electrophoresis and protein transfer. Samples were then run at 150 V for 1–2 h depending on the size of protein to be detected. Upon completion of electrophoresis, protein bands were transferred at 100 V for 2 h to Immobilon-P PVDF membrane (Millipore, #IPVH00010) using the NuPAGE Transfer Buffer (Invitrogen, #NP0006-1) with 10%–20% (v/v) methanol added. Resulting membranes would first be stained with Ponceau Red to ensure an even protein loading and transfer across all lanes, before blocking at RT for 1 h in 5% skim milk made up in 1X TBS pH 7.4 (Nacalai Tesque, #12748-31) with 0.05% (v/v) Tween-20 (TBS-T). A primary antibody for ACE2 (R&D, #AF933) was generally diluted with 5% skim milk in TBS-T and incubated at 4°C overnight, and secondary antibodies were diluted with 5% skim milk in TBS-T and incubated at RT for 1 h. Protein bands were then detected by using the ECL Primer Western Blotting Detection Reagent (Amersham, #RPN2232) and exposed using the ImageQuant LAS4000 biomolecular imager (Amersham).

### Virus infection and titration

For infection studies, SARS-CoV-2 Japan/TY/WK-521/2020 strain was confirmed by genome sequencing and used for infection studies. SARS-CoV-2 infection and titration were performed under BSL3 containment conditions. Airway epithelial cells and lung alveolar epithelial cells in ALI culture were infected with SARS-CoV-2 from the apical surface (10,000 PFU/well). Following 1 h incubation at 37°C, innoculum was removed and replaced with medium after three washes. Cells were incubated at 37 °C and 5% CO_2_ for 48 h. Cells containing supernatant were collected and stored at −80°C until analysis.

To determine the infectious virus titers, monolayers of VeroE6 cells constitutively expressing TMPRSS2 (VeroE6/TMPRSS2) (Matsuyama et al., 2010) were infected with serially diluted samples for 1 h incubation at 37°C, overlaid with minimum essential media containing 1% agarose, and incubated for 3 days at 37°C. After 3 days, plaques were visualized using 0.01% neutral red.

### Statistics

Microsoft Excel (version 2304), GraphPad Prism (version 9.5.0), and R 4.1.2 were used for data collection and analysis. We employed DESeq2 version 1.34.0 with a default parameter to perform statistical analyses on our bulk RNA-Seq data, which was based on raw counts. DESeq2 uses the Wald test to generate *p*-values, and these *p*-values are then adjusted for multiple testing using the Benjamini and Hochberg method as default. One-way ANOVA with Dunnett’s or Tukey’s multiple comparisons *post hoc* test was used to evaluate statistical significance when comparing more than two groups in infection data. The statistical significance level α was set to 0.05 for all experiments. Exact *p*-values are shown in the individual graphs.

### Electron microscopy

The airway and lung epithelial cells infected with SARS-CoV-2 were fixed with 2.5% glutaraldehyde in 0.1 M cacodylate buffer and postfixed with 1% osmium tetroxide. The samples were dehydrated with a gradient series of ethanol, substituted with propylene oxide, and embedded in epoxy resin. Ultrathin sections were stained with uranyl acetate and lead citrate and observed using a Hitachi HT-7700 microscope at 80 kV.

## Results

### ACE2 knockout by MMEJ-based guide RNA design

**FIGURE 1:**
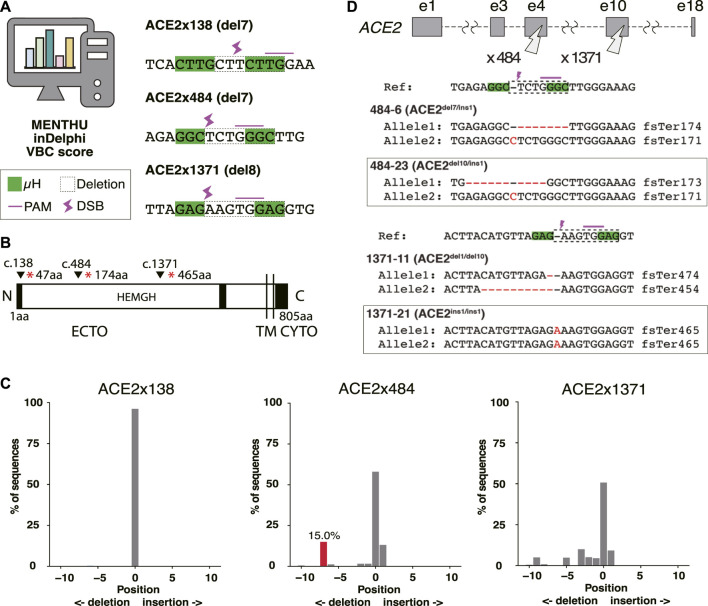
Genome editing strategy for ACE2 KO in B2-3 cells. **(A)** Designs of guide RNAs (gRNAs) used in this study. The PAM sequences are shown by the pink line over bases. Microhomologies (µHs) are boxed in green. The expected deletion is framed by the dotted line. **(B)** Diagram of ACE2 protein. The positions of each gRNAs are shown above the diagram. **(C)** Tracking of Indels by Decomposition (TIDE) analysis to identify the gene editing outcomes of bulk populations. **(D)** Sequences of each allele of the selected clones.

The ACE2 cell surface protein has been proposed to be essential for infection of human lung tissue by SARS-CoV-2 (Wang et al., 2022b). To model lung infection *in vitro*, we aimed to derive bronchial airway and alveolar epithelial cells from normal and gene-edited human iPS cells. To produce ACE2 KO iPS cell lines, gRNAs were designed to target Predominant MMEJ Allele (PreMA) sites using MENTHU (Figure 1A). Twelve gRNA candidates predicted to result in out-of-frame deletions with a MENTHU score >2.0 were identified (Supplementary Table S3). The expected MMEJ deletions were consistent with an alternative prediction algorithm, inDelphi (Shen et al., 2018). Additionally, their likelihood to result in loss-of-function mutations was further assessed using the Vienna Bioactivity CRISPR (VBC) score (Michlits et al., 2020). The VBC output auto-pick placed two of the top 10 gRNAs in exon 5, which also includes gRNA ACE2x484 (Supplementary Table S4). With these considerations, we selected 3 gRNAs: ACE2x138, ACE2x484, and ACE2x1371, to continue with gene editing experiments (Figures 1A, B).

The majority of the extracellular region of ACE2 (ECTO) is composed of a HEXXH zinc metallopeptidase domain (19aa–611aa) (Towler et al., 2004). The remainder of the protein is 48% identical to human collectrin (617aa–770aa) (Zhang et al., 2001) and contains a transmembrane domain (TM, 741aa–761aa) (Lambert et al., 2005) and cytoplasmic domain (CYTO) (Prabakaran et al., 2004). The three selected gRNAs disrupt the protein coding region prior to the catalytically active HEMGH domain, as well as the transmembrane (TM) anchoring domain (Jackson et al., 2022). ACE2 amino acids K^353^, K^31^, D^30^, D^355^, H^34^, D^38^, Q^24^, T^27^, Y^83^, Y^41^, and E^35^ are reported to be key residues to interact SARS-CoV-2 spike receptor-binding domain (Jawad et al., 2021). Therefore, MMEJ-induced frameshifts in these protein regions were expected to not only disrupt cell surface presentation and protein function but also exclude the spike protein interacting domain (353-357aa) known to be critical for viral interaction with ACE2 (Hayashi et al., 2020).

We evaluated gRNAs ACE2x138, ACE2x484, and ACE2x1371 for KO activity in the B2-3 lung reporter iPS cell line (Gotoh et al., 2014). Gene editing outcomes were confirmed by Sanger sequencing and TIDE analysis (Figure 1C). While ACE2x138 did not demonstrate any detectable indels with this assay, ACE2x484 and ACE2x1371 both showed indel formation (42% and 49.3%, respectively). In the ACE2x484 polyclonal population, 15% of indel alleles were represented by the predicted del7 mutation, while for ACE2x1371 the predicted del8 mutation was represented only 0.8% out of the total population. For both gRNAs, ins1 mutations were observed in the TIDE data (13.1% and 9.2%, respectively). The ACE2x484 gRNA had the highest MENTHU score in exon 5. It also ranked fourth when evaluated using the VBC score and was the second best based on the BioScore out of 13 gRNA on exon 5 (Supplementary Table S4).

**FIGURE 2:**
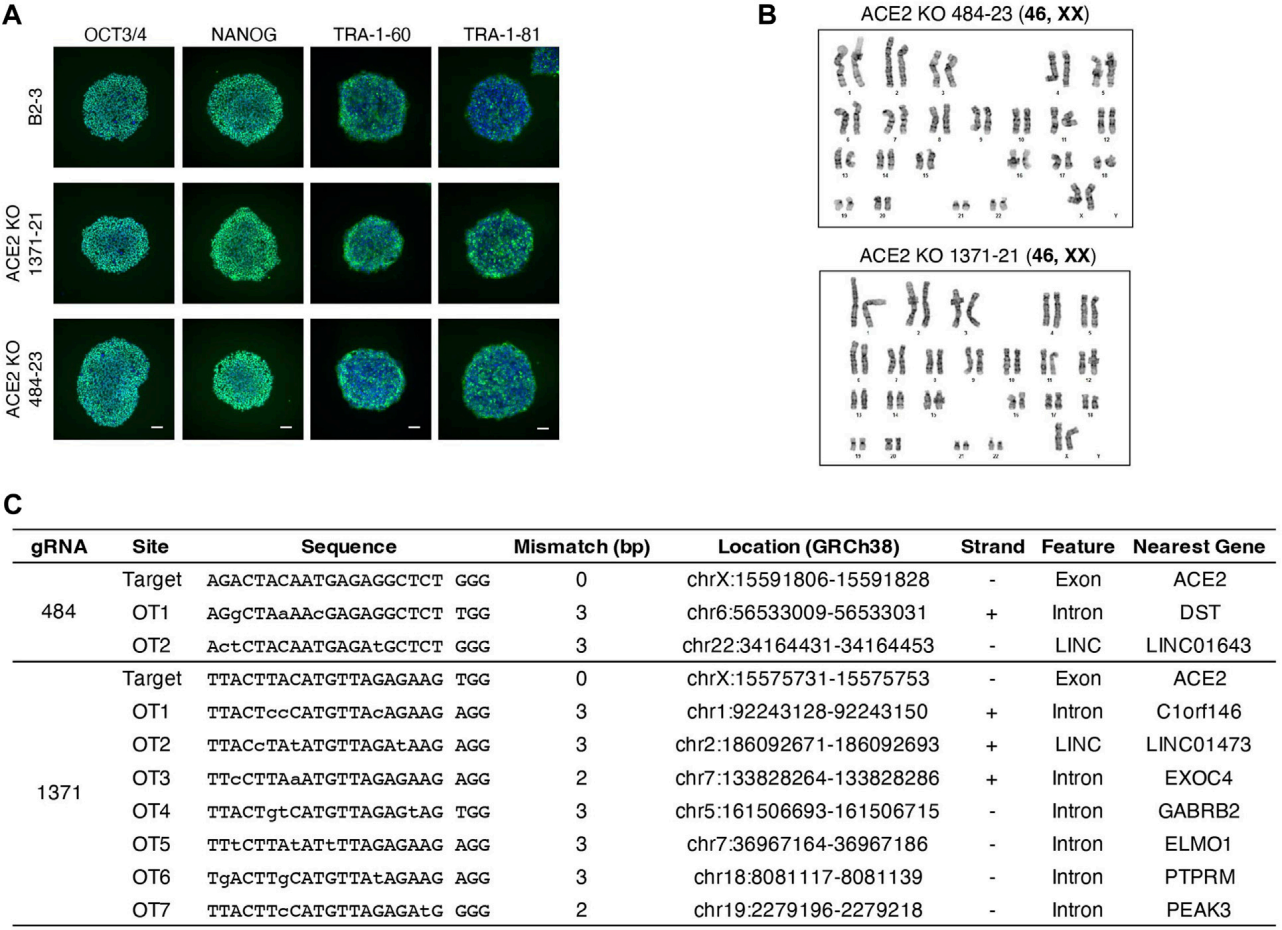
Quality control of ACE2 KO iPSC clones. **(A)** Immunostaining of pluripotency markers, OCT3/4, NANOG, TRA-1-60, and TRA-1-81. Scale bar 100 µm. **(B)** The image of karyotyping assay of 4 clones **(C)** Off-target sites were identified by GGGenome and were confirmed by Sanger sequencing to be unedited.

We generated clonal cell lines from ACE2x484 and ACE2x1371 edited populations (Figure 1D). All cell lines except 1371-21 were compound heterozygotes. For each allele bearing an indel, the predicted outcome was a frameshift resulting in the generation of a premature stop codon (Figure 1D). The clonal iPS cell lines 484-23 and 1371-21 were selected for subsequent experiments. The pluripotency of each iPS cell line was confirmed by immunostaining of OCT3/4, NANOG, TRA-1-60, and TRA-1-81 (Figure 2A). Karyotype (Figure 2B) and off-target analysis (Figure 2C) were performed, and no abnormalities were observed. These results indicate that DNA repair outcomes generated by CRISPR-Cas9 gene editing may be predicted based on sequence context and used to bias the resulting KO alleles.

### Differentiation of ACE2 KO iPS cells into lung airway and alveolar epithelia

**FIGURE 3:**
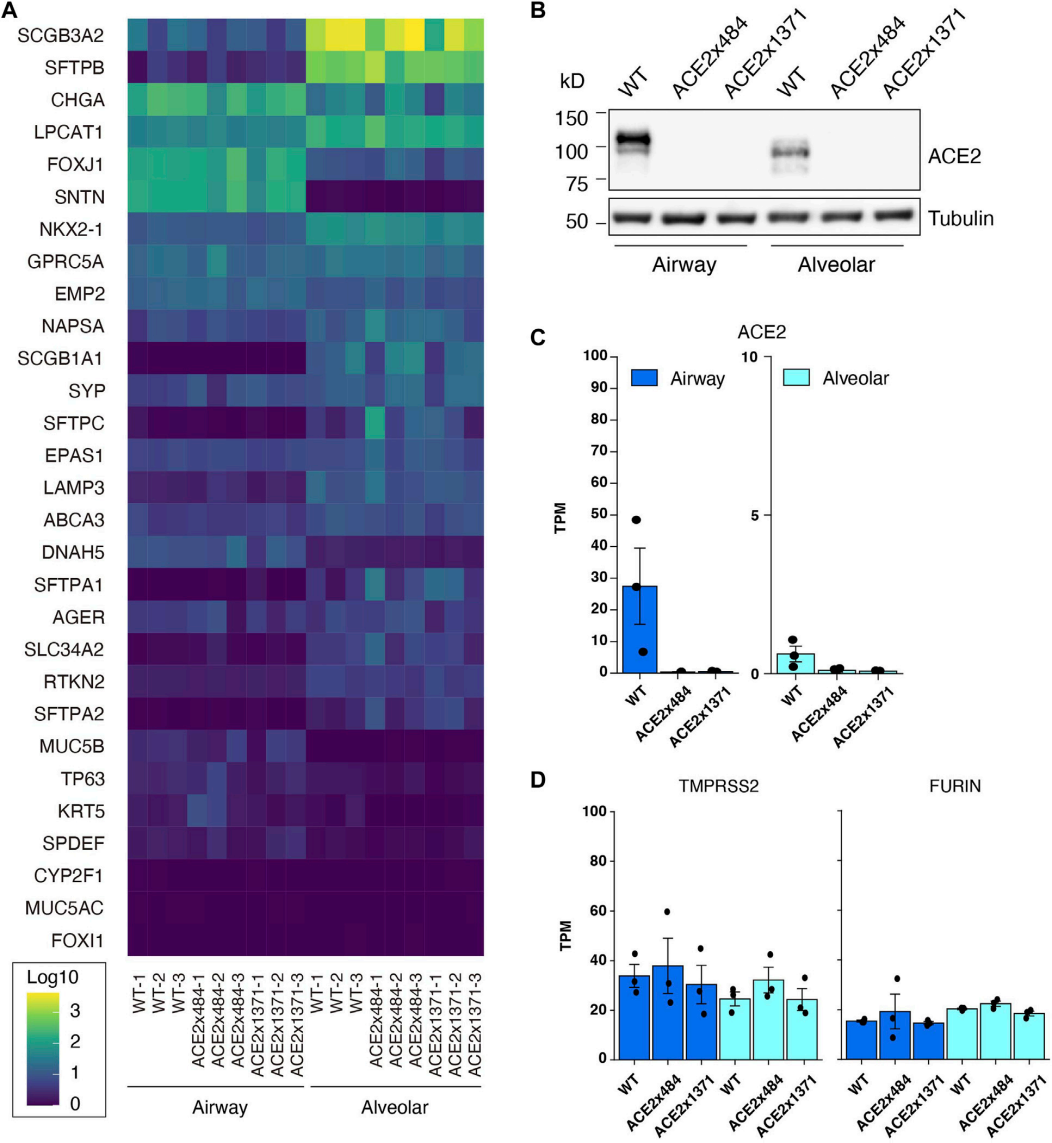
Confirmation of iPS cell differentiation and ACE2 KO. **(A)** RNA-Seq to quantify the expression of lung-related genes in differntiated ACE2 WT and KO iPSCs (Log10 TPM). **(B)** Western blotting for ACE2 in WT and KO cells. **(C)** RNA-Seq analysis showing reduced expression of ACE2 mRNA in KO cells. **(D)** Expression of proteases related to viral infection remain unchanged. The dots in the bar graphs represent biological replicates (N = 3). No statistically significant difference was observed in the expression level of differentiation markers or proteases between differentiated WT and ACE2 KO cells (DESeq2 adjusted *p*-value >0.05).

ACE2 KO iPSC clones 484-23 and 1371-21 were differentiated into airway epithelial and alveolar cells (Gotoh et al., 2014), and evaluated by RNA Sequencing (RNA-Seq) for the expression of lung-tissue related genes (Figure 3A). Upon comparison, the knockout cell lines did not exhibit substantial differences in gene expression relative to the parental B2-3 cell line. Expression of ACE2 in parental and ACE2 KO iPSC lines was evaluated by Western blot and RNA-Seq following differentiation (Figures 3B, C). ACE2 protein could be detected in airway cells and at a lower level in alveolar epithelial cells but was undetectable in both lung cell lineages derived from ACE2 KO iPS cells edited using the x484 or x1371 gRNAs. Additional genes associated with SARS-CoV-2 infection, namely, *FURIN* and *TMPRSS2*, were confirmed to be expressed at similar levels in WT and KO cells (Figure 3D). Moreover, the expression of various type II transmembrane serine proteases (TTSP) including TMPRSS11A and TMPRSS11D remained unchanged between WT and ACE2 KO cells (Supplementary Table S5). Based on these data, we concluded that ACE2 KO does not affect lung differentiation *in vitro* or the expression of other genes associated with SARS-CoV-2 infection.

### ACE2 KO reduces SARS-CoV-2 replication

**FIGURE 4:**
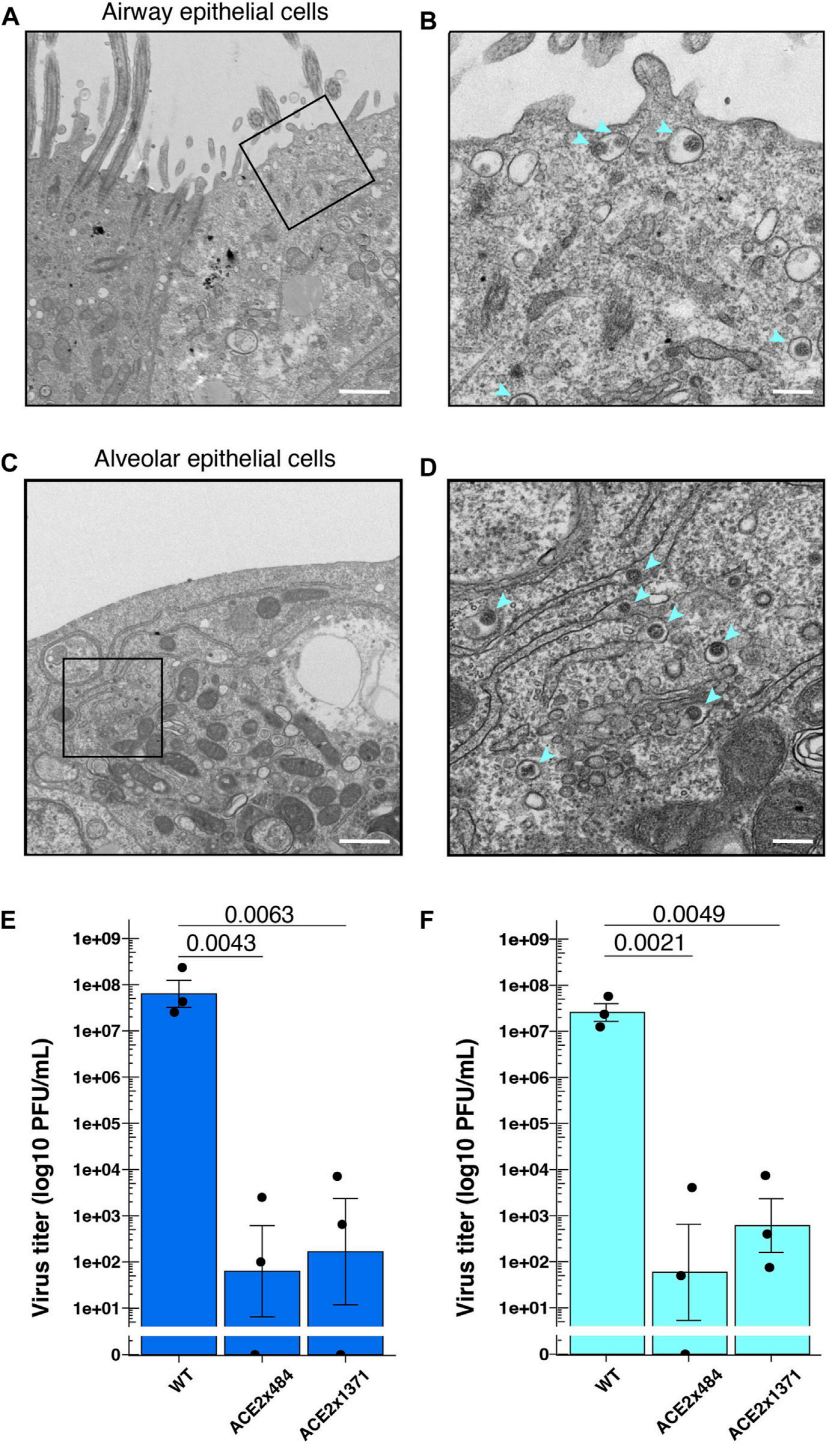
SARS-CoV-2 infection in the gene edited iPS cell lung model. **(A–D)** Observation of SARS-CoV-2 viral particles in wild-type airway and alveolar epithelial cells using TEM. The images were taken at 4 dpi (days post-infection) in airway epithelial cells **(A,B)** and 2 dpi in alveolar epithelial cells **(C,D)**. A and C were imaged at ×5,000 magnification with a 1 µm scale bar, while B and D were further enlarged to a scale equivalent of ×20000 magnification with a 200 nm scale bar. **(E)** Measurement of virus titers following infection of SARS-CoV-2 strain WK521 into parental and ACE2 KO iPS cell-derived airway and **(F)** alveolar epithelial cells. *p*-value from one-way ANOVA with Dunnett’s or Tukey’s multiple comparisons *post hoc* test is shown in the individual graphs. The dots represent each biological replicate (N = 3).

Next, we examined SARS-CoV-2 replication in differentiated airway and lung epithelial cells to evaluate the suitability as *in vitro* virus infection models. SARS-CoV-2 strain WK521 virions were observed within both airway and alveolar cells (Figures 4A–D). We next infected WT and ACE2 KO lung cells *in vitro* and measured viral titer to quantify the impact of ACE2 KO on viral replication. SARS-CoV-2 strain WK521 replicated efficiently in both WT airway and alveolar epithelial cells derived from parental B2-3 iPS cells (Figures 4E, F). In contrast, ACE2 KO using ACE2x484 or ACE2x1371 resulted in the reduction of SARS-CoV-2 replication in the airway and alveolar epithelial cells. These data demonstrate that ACE2 expression is essential for SARS-CoV-2 replication in iPS cell-derived airway and lung cells, thereby validating our model system.

## Discussion

**FIGURE 5:**
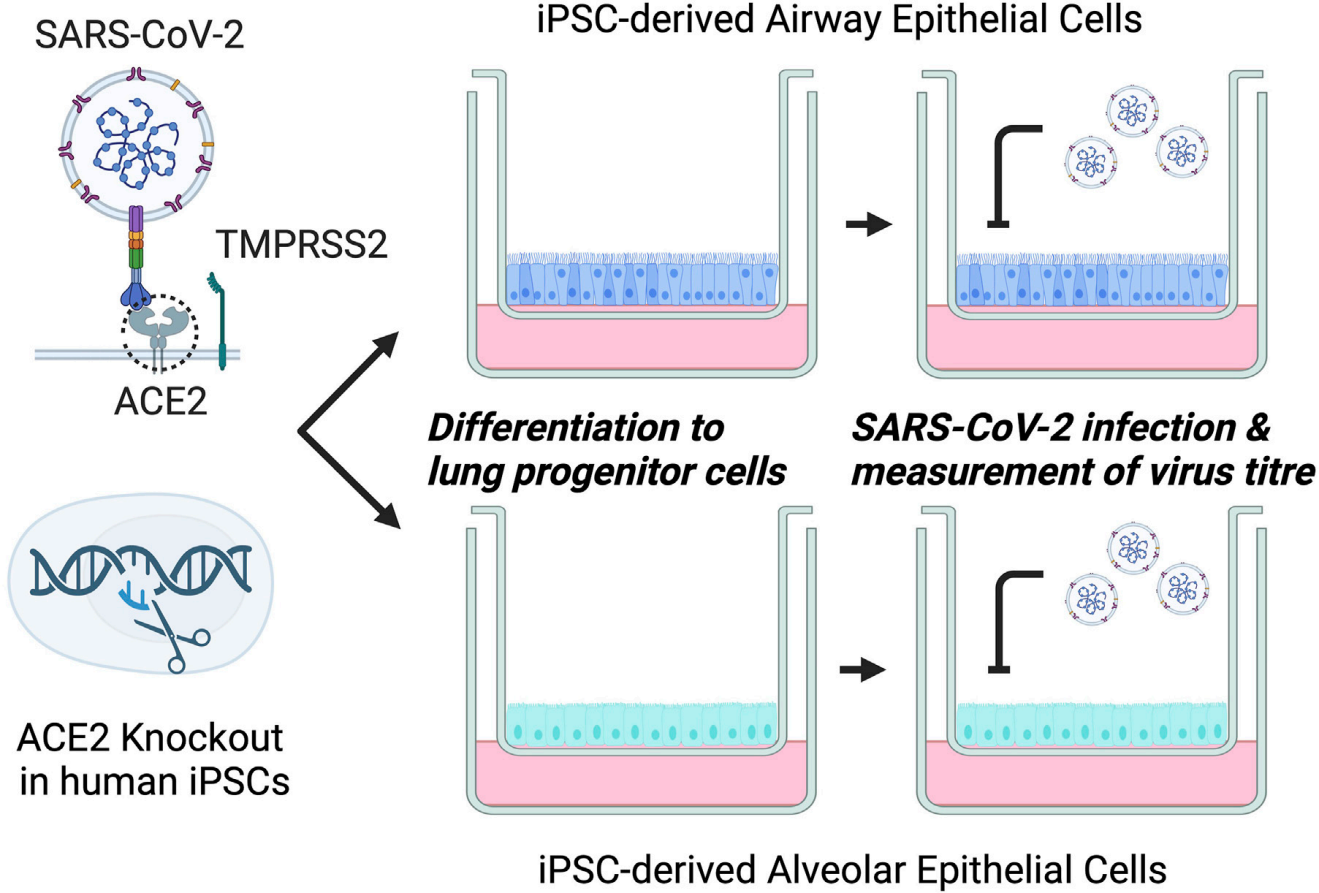
Workflow of this study.

Here, we showcase the utility of *in silico*-based gRNA design to bias the generation of gene KOs in human iPS cells. Additionally, we establish a model system based on iPS cell-derived lung cells and show that they are susceptible to infection by common SARS-CoV-2. Subsequently, we use this genetically engineered model system to demonstrate that ACE2 KO hinders SARS-CoV-2 viral replication, verifying an essential pathway for SARS-CoV-2 viral entry *in vitro* (Figure 5).

In this study, MMEJ was used for gene knockout. However, of the three gRNAs tested in this study, only ACE2x484 produced the predicted PreMA alleles at high frequency. Various tools to predict gene editing outcomes have been proposed in recent years (Nakamae and Bono, 2022). Tools that predict gRNA activity using linear regression and machine learning models provide reliable gRNAs and reproducible gene editing outcomes to streamline the production of engineered cell lines (Konstantakos et al., 2022). Our goal for ACE2 editing was to employ MMEJ-induced deletions, resulting in predictable frameshifts leading to gene KO. MENdel is effective for predicting MMEJ and single nucleotide insertions and can achieve better accuracy in designing gRNAs (Martínez-Gálvez et al., 2021). While empirical testing of gRNAs in the target cell line is ultimately required, the data presented here shows that *in silico* prediction can help streamline gene editing projects.

ACE2 is an essential component for SARS-CoV infection (Matsuyama et al., 2010). A role of ACE2 for *in vitro* SARS-CoV-2 infection has been previously revealed in human A549 lung cancer cells and African green monkey Vero-E6 kidney cells using CRISPR screening (Daniloski et al., 2021; Wei et al., 2021). An *in vitro* model using kidney differentiation demonstrated that ACE2 is required for SARS-CoV-2 infection (Helms et al., 2021). Similarly, we used *in vitro* iPSC differentiation into two major lung cell types to study lung infection. Using RNA-Seq and WB we confirmed that ACE2 expression is higher in airway epithelial cells than alveolar epithelial cells, consistent with primary cells from human donors (Ziegler et al., 2020). Interestingly, a shift in ACE2 molecular weight between cell types was also observed in our *in vitro* model, suggesting that differential glycosylation may occur, as previously observed in human bronchial epithelial cells (Shajahan et al., 2021). Finally, the iPS cell lines generated in this study confirm that ACE2 KO does not significantly alter lung differentiation, providing an important resource for studying SARS-CoV-2 infection with *in vitro* generated lung tissue.

We acknowledge several limitations of the current study that open avenues to future research. Firstly, our work was conducted using a single iPS cell line, B2-3. Future studies would benefit from utilizing multiple iPS cell lines, in particular COVID-19 patient-derived iPS cell lines to explore the role of human genetic variants in SARS-CoV-2 infection susceptibility and severity. Similarly, we only investigated one strain of the virus (WK521). Since the start of the COVID-19 pandemic, SARS-CoV-2 infections have given rise to multiple genetic variants with S-protein mutations (Cox et al., 2023). Our *in vitro* system should prove valuable for screening viral variants for their infectious properties and host responses, including the emerging variants of concern. Finally, our investigation was limited to two cell types. ACE2 KO iPS cells may be differentiated into other cell types that express ACE2 and may be susceptible to infection such as cardiac cells (Hikmet et al., 2020). Conversely, ACE2 KO iPS cells could aid in our understanding of ACE2-independent pathways of viral entry. Prospective studies using differentiated ACE2 KO iPS cells should reveal the multifaceted interactions between SARS-CoV-2 and host cells.

The COVID-19 pandemic demonstrated that an understanding of individual susceptibility to infectious disease is needed to identify risk groups and develop relevant interventions. Several reports suggested that human genetic variants are associated with the susceptibility to infection and the severity of COVID-19 (Zeberg and Pääbo, 2020; Niemi et al., 2021). For example, previous reports suggest that immune-mediated inflammation plays a major role in the symptoms of COVID-19 (Tay et al., 2020; Cheon et al., 2021). The construction of *in vitro* experimental models is essential to advance research that may reveal such genetic variants. Through ACE2 KO, we showed the possibility of combining gene editing and infection models to explore the phenotypes of infectious disease. The presence of low doses of infectious virus in ACE2 KO iPS cell-derived airway epithelial cells and lung alveolar epithelial cells suggests either residual inoculated virus or viral replication by ACE-independent cell entry pathway. Beyond the role of ACE2 targeted in this study, it will be important to reveal the necessity for other genes implicated in SARS-CoV-2 infection including CATHEPSIN, TMPRSS2, and FURIN through reverse genetics.

Collectively, the current study using iPS cell-derived lung cells provide insights into the mechanism of viral infection, which may lead to the development of anti-viral therapies and a deeper understanding of the molecular biology behind COVID-19.

## Data Availability

The datasets presented in this study can be found in online repositories. The names of the repository/repositories and accession number(s) can be found below: https://www.ncbi.nlm.nih.gov/, PRJDB15620.
