# Corrigendum: ACE2 knockout hinders SARS-CoV-2 propagation in iPS cell-derived airway and alveolar epithelial cells

**DOI:** 10.3389/fcell.2024.1407164

**Published:** 2024-06-21

**Authors:** Ryo Niwa, Kouji Sakai, Mandy Siu Yu Lung, Tomoko Matsumoto, Ryuta Mikawa, Shotaro Maehana, Masato Suzuki, Yuki Yamamoto, Thomas L. Maurissen, Ai Hirabayashi, Takeshi Noda, Makoto Kubo, Shimpei Gotoh, Knut Woltjen

**Affiliations:** ^1^ Department of Life Science Frontiers, Center for iPS Cell Research and Application (CiRA), Kyoto University, Kyoto, Japan; ^2^ Graduate School of Medicine, Kyoto University, Kyoto, Japan; ^3^ Department of Veterinary Science, National Institute of Infectious Diseases, Tokyo, Japan; ^4^ Management Department of Biosafety, Laboratory Animal and Pathogen Bank, National Institute of Infectious Diseases, Tokyo, Japan; ^5^ Department of Clinical Application, Center for iPS Cell Research and Application (CiRA), Kyoto University, Kyoto, Japan; ^6^ Department of Microbiology, Kitasato University School of Allied Health Sciences, Kanagawa, Japan; ^7^ Regenerative Medicine and Cell Design Research Facility, Kitasato University School of Allied Health Sciences, Kanagawa, Japan; ^8^ Antimicrobial Resistance Research Center, National Institute of Infectious Diseases (NIID), Tokyo, Japan; ^9^ Laboratory of Ultrastructural Virology, Institute for Life and Medical Sciences, Kyoto University, Kyoto, Japan; ^10^ Laboratory of Ultrastructural Virology, Graduate School of Biostudies, Kyoto University, Kyoto, Japan

**Keywords:** iPS cells, CRISPR-Cas9, gene editing, gene knockout, ACE2, SARS-CoV-2

In the published article, there was an error. The **Results** section regarding the reported proportion of DNA sequence outcomes for one gRNA contained a mistake. While the data in the associated figure was correct, an error in the main text was identified during a subsequent review of our data.

A correction has been made to **Results**, *ACE2 knockout by MMEJ-based guide RNA design*, Paragraph 3. This sentence previously stated:

“We evaluated gRNAs ACE2x138, ACE2x484, and ACE2x1371 for KO activity in the B2-3 lung reporter iPS cell line (**Gotoh et al., 2014**). Gene editing outcomes were confirmed by Sanger sequencing and TIDE analysis (**Figure 1C**). While ACE2x138 did not demonstrate any detectable indels with this assay, ACE2x484 and ACE2x1371 both showed indel formation (42% and 60.1%, respectively). In the ACE2x484 polyclonal population, 15% of indel alleles were represented by the predicted del7 mutation, while for ACE2x1371 the predicted del8 mutation was represented only 3.9% of the total population. For both gRNAs, ins1 mutations were observed in the TIDE data (14.7% and 39.9%, respectively). The ACE2x484 gRNA had the highest MENTHU score in exon five and is also supported by being in the top four in VBC score top two in BioScore (**Supplementary Table S4**).”

The corrected sentence appears below:

“We evaluated gRNAs ACE2x138, ACE2x484, and ACE2x1371 for KO activity in the B2-3 lung reporter iPS cell line (**Gotoh et al., 2014**). Gene editing outcomes were confirmed by Sanger sequencing and TIDE analysis (**Figure 1C**). While ACE2x138 did not demonstrate any detectable indels with this assay, ACE2x484 and ACE2x1371 both showed indel formation (42% and 49.3%, respectively). In the ACE2x484 polyclonal population, 15% of indel alleles were represented by the predicted del7 mutation, while for ACE2x1371 the predicted del8 mutation was represented only 0.8% out of the total population. For both gRNAs, ins1 mutations were observed in the TIDE data (13.1% and 9.2%, respectively). The ACE2x484 gRNA had the highest MENTHU score in exon 5. It also ranked fourth when evaluated using the VBC score and was the second best based on the BioScore out of 13 gRNA on exon 5 (**Supplementary Table S4**)”.

The authors apologize for this error and state that this does not change the scientific conclusions of the article in any way. The original article has been updated.

